# RAMIE: tradition drives innovation—feasibility of a robotic-assisted intra-thoracic anastomosis

**DOI:** 10.1007/s13304-020-00932-1

**Published:** 2020-11-27

**Authors:** Simone Giacopuzzi, Jacopo Weindelmayer, Giovanni de Manzoni

**Affiliations:** General and Upper GI Surgery Division, Azienda Ospedaliera Universitaria Integrata Verona, Piazzale Aristide Stefani 1, 37126 Verona, Italy

**Keywords:** RAMIE, Ivor lewis, Robotic surgery

## Abstract

Due to the difficulties in the intra-thoracic esophagogastric anastomosis creation, totally minimally invasive Ivor Lewis esophagectomy (MIE) did not encountered a large diffusion, preferring hybrid techniques or cervical anastomosis. Robot-assisted minimally invasive esophagectomy (RAMIE) has gained popularity due to an easy reproducibility of the open anastomotic technique. In this feasibility study, we described the RAMIE technique introduced in our Center, providing innovative details for a mechanical end-to-end anastomosis. With patient in prone position, esophagectomy is conducted through the meso-esophagus plan. Robotic hand-sewn purse-string is realized above Azygos vein. A 4-cm thoracotomy in the fifth intercostal space is performed by enlarging the trocar incision. The tubulization is performed to create an access pouch for the introduction of the circular stapler. After the creation of the end-to-end anastomosis, the access pouch is resected and a robotic over-sewn is realized. From January 2020 until July 2020, ten patients were enrolled. No restriction in term of age, BMI, ASA grade or previous surgery were applied. Median operative time was 700 min. R0 resection was achieved in all cases with a good lymph node harvesting. No anastomotic leak or stricture were observed. One chyle leak was treated conservatively. Median length of stay was 8 days and 90 days mortality was 0%. This study evidenced how robotic surgery allowed us to perform the same anastomosis of our open technique with good oncological results and morbidity and length of stay comparable with our previous results. Of note, longer operative time has been recorded. Further studies after the completion of the learning curve are necessary to address more definite conclusions.

## Introduction

A growing incidence of distal and gastroesophageal junction tumors in Western countries have pushed esophageal surgeons towards the two field Ivor Lewis esophagectomy and, in the last few years, it has become the preferred procedure in many high volume Centers [1].

Despite surgical advancements, esophagectomy remains a high-risk operation, with a 60% morbidity and a 4.5% mortality and, among all the complications, anastomotic leak remains one of the most feared with an incidence of about 10% [2]. Due to the difficulties encountered in the thoracic creation of the esophagogastric anastomosis, totally minimally invasive Ivor Lewis esophagectomy (MIE) with laparoscopic and thoracoscopic approaches did not encounter a large diffusion, with the majority of surgeons performing a hybrid laparoscopic-thoracotomic esophagectomy (HE). Notably, this HE approach led to a reduction in pulmonary complications in the French randomized MIRO trial [3]. Unfortunately, the attempts to perform a totally MIE with a thoracoscopic approach have led to an increase of anastomotic leak up to 15–20% [4, 5].

Together with the advancement of the robotic technology in surgery, robot-assisted minimally invasive esophagectomy (RAMIE) has gained popularity due to an increased precision in the mediastinal dissection and an easy reproducibility of the open anastomotic technique as the robot guarantee a 3D imaging of superior quality and free articulation of the instruments. The German group standardized the RAMIE technique in 2019 and more recently, a European study on 100 patients comparing RAMIE with conventional MIE evidenced a reduction in ICU stay with a trend for a better lymphadenectomy for RAMIE procedure [6, 7].

In this feasibility study, we described the RAMIE technique introduced in our Center since January 2020, highlighting the advantages of the robotic approach in the mediastinal dissection and in the anastomotic technique, providing innovative details for a mechanical end-to-end anastomosis.

## Surgical technique

### Abdominal phase

To focus on the thoracic phase of the esophagectomy and considering the standardization of the technique in our Department, in this early experience, we decided to perform the abdominal part laparoscopically.

**Fig. 1:**
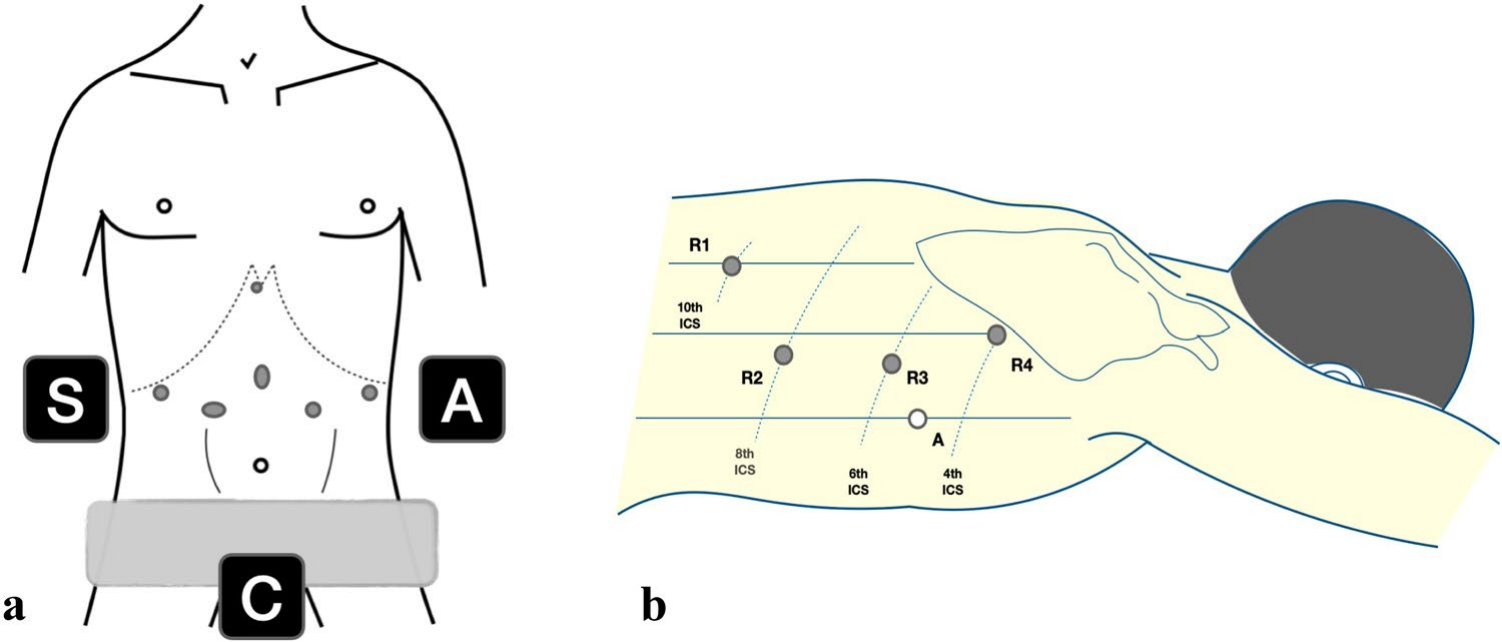
**a** Abdominal port placement and disposition of surgeons in the abdominal phase (S: surgeon, first operator, *A* assistant, *C* camera operator). **b** Thoracic port placement in prone position (*R* robot arm, *A* assistant port, *ICS* intercostal space)

**Fig. 2:**
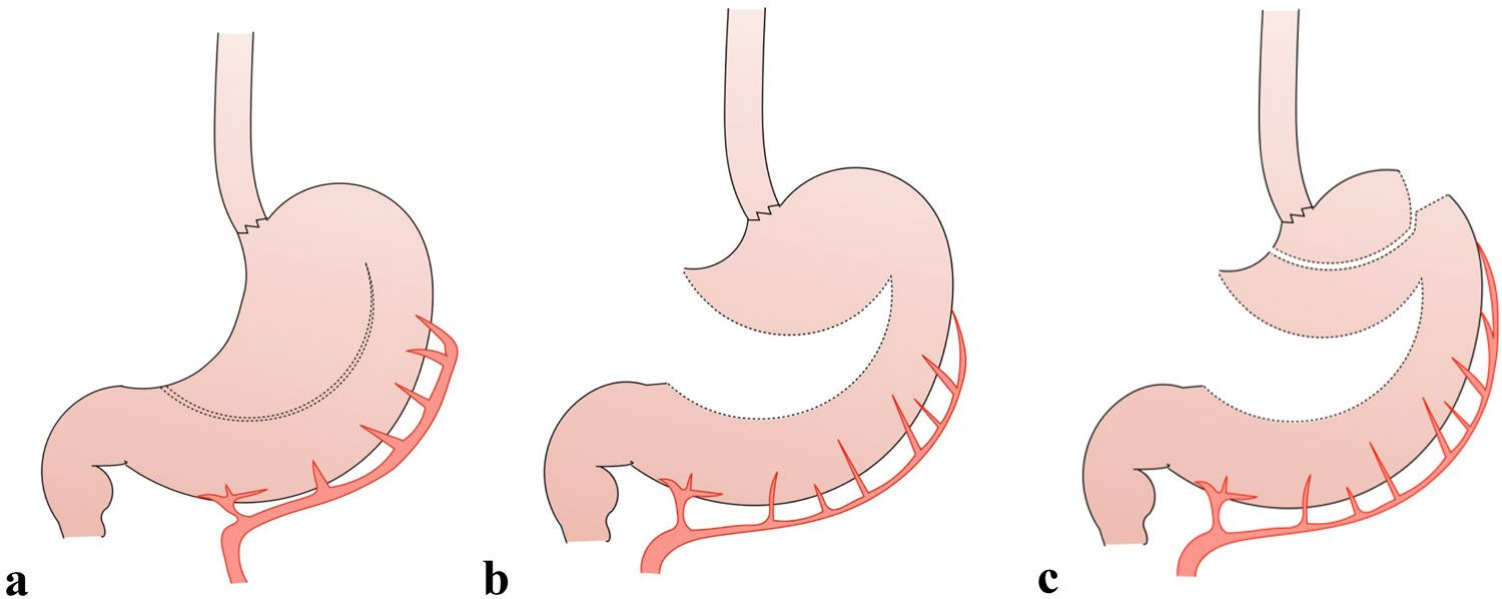
**a** Abdominal gastric tubulization, **b** abdominal gastric tubulization, and **c** thoracic completion of tubulization

The following steps for the abdominal dissection are performed with the patient in French position. The camera port is inserted halfway between the xiphoid and the umbilicus. The other ports are placed to create a W-shaped line (Fig. 1a). The surgeon stands at the right side of the patient, the assistant at the left side and camera operator between patient’s legs. The liver is lifted using a Nathanson Liver Retractor (Storz, Germany) inserted in the subxiphoidal space. With the assistant lifting the stomach and the omentum, the operator performs a dissection of the gastrocolic ligament, in a caudo-cranial direction, 2–3 cm distant from the gastroepiploic vessels. Lesser omentum is then dissected close to the liver and the right gastric artery is identified and divided. The next step is the tubulisation of the distal part. The stomach is first sectioned obliquely using a 45-mm linear stapler starting from 2 cm above the pylorus towards the greater curvature, and then parallel to this, using one 60-mm linear stapler, to obtain a 30-mm-wide gastric conduit (Fig. 2a, b). This maneuvre allows a wider view on the left gastric vessels. En bloc D2 dissection through the hepatic ligament, the celiac trunk and the splenic vessels is then performed, and the left gastric vessels are ligated.

The phrenoesophageal membrane of Laimer-Bertelli is opened and the esophagus is encircled by an umbilical tape. A small section on the left side of the diaphragmatic crura is usually performed to ensure a more effective access to the lower mediastinum. Mediastinal esophageal dissection is carried out for 4–5 cm in the lower mediastinum. Last step is completion of abdominal tubulisation with more 60-mm linear staplers parallel to the greater curve (Fig. 2a, b).

### Thoracic robot-assisted phase

Patient position shown in Fig. 1b is the modified prone position and ports are placed as described by Egberts in 2019 [6].

Esophagectomy is conducted through the dissection plan of the meso-esophagus, including the thoracic duct in the en bloc resection. Identification of the duct is facilitated using the robotic nearly-infrared camera (Firefly, Intuitive surgical, USA) after injecting indocyanine green in the inguinal lymph nodes at the end of the abdominal phase [8].

After complete mobilization of the esophagus and division of Azygos vein, a robotic hand-sewn purse-string is realized in the esophagus above the level of the vein. A 4-cm thoracotomy in the fifth intercostal space is then performed by enlarging the incision of the assistant trocar. The anvil of a 25-mm circular stapler is inserted through the thoracotomy in the esophageal lumen. Afterwards, the specimen is pulled out and the tubulization is completed: the 30-mm-large conduit tip is realized using a linear stapler inserted from the fundus and directed caudally parallel to the greater curvature. A section in the direction of the lesser curve completes the creation of the access pouch that will be used to introduce the circular stapler (Fig. 2c). After assessing the optimal length of the conduit, the tip is resected at the selected level with a linear stapler placed at an oblique angle (longer in the mesenteric/shorter in the antimesenteric side).

**Fig. 3:**
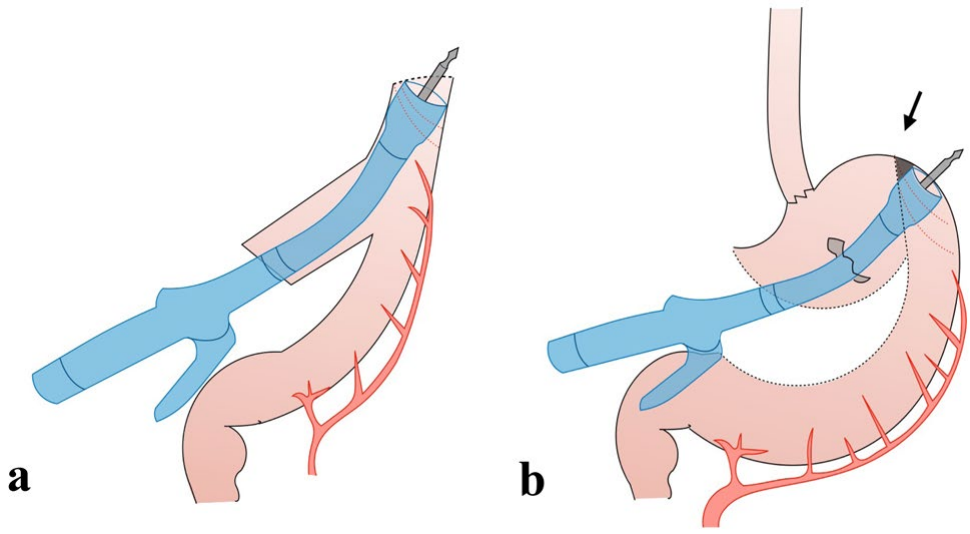
**a** Gastric conduit with the circular stapler inserter through the access pouch. The tip of the stapler comes closer to the greater curve edge. This maneuvre together with the oblique section of the conduit’s tip results in the formation of one small anterior dog-ear that will be covered with the anastomotic over-sewn. **b** The classic anastomosis on the posterior wall results in a small ischemic area on the antimesenteric side (black arrow)

The shape of the conduit allows an easy introduction of the circular stapler and a safe execution of the anastomosis. Moreover, the suture thus achieved, (as previously described for open technique by our group [9], is a real end-to-and anastomosis and could avoid the theoretical ischemic area of anastomoses realized on the greater curvature (Fig. 3). The access pouch is then resected using 1 or 2 linear staplers introduced from the tenth intercostal space trocar. The final step is the robotic over-sewn of the circular staple line using a barbed suture.

## Patients and methods

We conducted a retrospective study on a prospectively maintained database including all the patients who underwent Ivor Lewis RAMIE from the beginning of our experience in January 2020 until July 2020. Of note, Covid-19 pandemic has slowed down the recruitment of the patients and the availability of the operating theatre.

Patients with a clinical infiltration of the periesophageal structures (cT4a) and patients with bulky nodes have been excluded from this primary experience. No restriction in term of age, BMI, ASA grade or previous surgery have been established.

All the operations have been carried out by two surgeons experienced in laparoscopic and thoracoscopic esophageal surgery (SG and JW). Both surgeons had basic robotic experience in upper gastrointestinal surgery for benign disease using the da Vinci Xi system (Intuitive surgical, USA).

All the patients have been included and treated according to our institutional ERAS program for esophagectomy [10].

Basic demographics, tumor and oncological characteristics, operative details and postoperative outcomes were recorded. Morbidity, mortality and readmission were assessed at 90 days, and complications were graded according to Clavien–Dindo classification.

Anastomotic and chyle leaks were defined and graded according to ECCG [1]. No routine assessment of anastomosis integrity was performed. Anastomotic leak was suspected on a clinical basis and investigated with CT scan with soluble contrast swallow and endoscopy.

## Results

A total of ten patients have been included in this very preliminary experience. Basic demographic and tumor features are described in Table 1. All the included patients were male and more than half of them were overweight or obese.

**Table 1:** Patients and tumor features

	*n* = 10
Sex	
Female	0
Male	10
Age, median (range)	66.5 (45–80)
BMI, median (range)	26 (21–36)
Comorbidity	
Smoking	7
Alcohol abuse	0
Cardiovascular	5
Respiratory	0
Metabolic	5
ASA	
1–2	7
3–4	3
Tumor histology	
Adenocarcinoma	9
Squamous cell carcinoma	1
Tumor location	
Middle esophagus	3
Lower esophagus	5
Esophagogastric junction	2
Neoadjuvant treatment	
No	1
Chemotherapy	2
Chemoradiotherapy	7

Histotype was adenocarcinoma of the esophagus or esophagogastric junction in nine of the ten patients and nearly all underwent neoadjuvant treatment before surgery.

Median operative time was 700 min and we could not observe any trend towards a time reduction, probably due to the small size of the cohort. Neither major adverse events nor conversion to open surgery were registered in the abdominal part while two patients required a conversion to open thoracic approach. At the very beginning of our robotic experience, we experienced a bleeding from the Azygos vein due to a wrong movement of one robotic arm. Urgent conversion was required to control the bleeding and the operation was then completed in open approach. In one patient, aortic adhesion of the primary tumor was discovered intraoperatively. Conversion to open surgery was, therefore, necessary to safely extend the resection along the aortic wall. Interestingly, these patients had a lower lymph node retrieval compared with the completely minimally invasive ones (Table 2).

**Table 2:** Intraoperative outcomes of the ten patients listed in chronological order

Patient	Operating time (min)	Conversion abdomen	Conversion thorax	Intraoperative complications	Blood transfusion	Resected nodes	pTNM
1	660	No	Yes	Bleeding (Azygos)	No	18	ypT0N1
2	720	No	No		No	32	ypT0N0
3	720	No	No		No	24	pT2N3
4	750	No	No		No	23	ypT2N1
5	600	No	Yes	Aortic adhesion	No	17	ypT0N0
6	780	No	No		No	24	ypT1bN0
7	680	No	No		No	26	ypT2N0
8	790	No	No		No	31	ypT1aN0
9	660	No	No		No	27	ypT0N0
10	600	No	No		No	24	ypT2N0

R0 resection was achieved in all cases with a good lymph node harvesting.

Our ERAS protocol for Ivor Lewis esophagectomy expect an immediate extubation and transfer to a surgical progressive care unit. Nevertheless, in this series, five patients were transferred to the intensive care unit (ICU) after the operation. One of them required a prolonged intubation due to a severe pulmonary distress while the others spent one night in ICU because the operation ended in late afternoon.

Six patients had a postoperative complication. One patient presented a chyle leak treated with dietary modifications.

Regarding the anastomosis, we did not observe anastomotic leak or stricture in this series. Nine patients resumed a soft diet on POD 4 (as scheduled in ERAS protocol) with a good compliance.

Only one patient presented a severe pulmonary complication that required a prolonged ICU monitoring and ventilatory support.

Median length of hospital stay of the whole cohort was 8 days and no patient died within 90 days after the operation (Table 3).

**Table 3:** Postoperative outcomes

	*n* = 10
ICU, median (range)	0.5 (0–17)
Readmission ICU	0
Complications	
Anastomotic leak	0
Chylothorax type Ia	1
Cardiac	1
Pulmonary	4
Most severe complication	
No	5
CD I	1
CD II	1
CD IIIa	2
CD IIIb	0
CD IV	1
LOS, median (range)	7.5 (6–25)
Readmission	1
90-day mortality	0
Radicality, R0	10
Resected lymph nodes, median (range)	24 (17–32)

*ICU* Intensive Care Unit; *CD* Clavien–Dindo classification; *LOS* Length of Stay

## Discussion

Minimally invasive approach in esophagectomy for cancer is gaining popularity in Western and Eastern series. However, evidences supporting its application are still weak and a great variability in the various MIE techniques (hybrid or totally MI) increases the difficulty in comparing the results between different series. Only two randomized trials have been conducted on MIE in the West. The French MIRO trial [3] compared the open approach with a hybrid technique (laparoscopic + thoracotomic), performing the anastomosis through a thoracotomy. On the other hand, in the TIME trial [11], the MIE was a McKeown operation with a cervical anastomosis. In both trials, the anastomosis was, therefore, carried out in a traditional manner, thus highlighting the problems in the standardization of a minimally invasive intra-thoracic anastomosis. This reluctance is based on the high leakage rate encountered in the few published experiences on totally MIE [5].

Nevertheless, Ivor Lewis procedure is the procedure of choice for the majority of esophageal cancer in the West, considering the high incidence of esophageal and esophagogastric junction adenocarcinoma [12].

In this scenario, the advantages brought by the robotic approach could assist the surgeon in the execution of a safe and “as close as possible to the standard open technique” anastomosis.

This feasibility study, aimed to use the robotic surgery to perform the same anastomosis described in our open technique [9]. The first ten patients had satisfactory oncological and clinical results, with an adequate number of lymph nodes harvested in all the patients and a morbidity and length of stay comparable with our recently published results [13]. Nonetheless, these results came at the price of a longer operative time and with half of the patients who spent one night in ICU for close monitoring. Transthoracic RAMIE requires a long learning curve and operative time are longer than hybrid or open surgery with operative time varying between 367 and 693 min at the beginning or the robotic experience [14] and reaching a median of 415 min in the largest European series [7]. Although considering the small sample size that does not allow to draw any reliable conclusion, we did not observe any anastomotic leak or stricture in our series. The largest paper on RAMIE including 100 patients reported a leak rate of 8% [7] and some other small preliminary experiences described even a higher incidence of this complication [15, 16].

The preliminary nature of the study aimed only to describe the technique and its safety and further data are required to assess its effectiveness in short- and long-term results; this case series does not represent a revolution, but it indicates a continuity with our history. We believe that this can be a good example on how the robotic approach can overcome some limitations of the laparoscopy allowing the surgeon to perform an operation without compromises.
